# Can the work ability model provide a useful explanatory framework to understand sustainable employability amongst general practitioners: a qualitative study

**DOI:** 10.1186/s12960-018-0292-x

**Published:** 2018-07-24

**Authors:** Jasmin Smyth, Sabrina Winona Pit, Vibeke Hansen

**Affiliations:** 1 University of Wollongong, School of Medicine, 61 Uralba Street, PO Box 3074, Lismore, New South Wales 2480 Australia; 2 Western Sydney University, University Centre for Rural Health, School of Medicine, 61 Uralba Street, PO Box 3074, Lismore, New South Wales 2480 Australia; 3 Sydney University, University Centre for Rural Health, School of Rural Health, 61 Uralba Street, PO Box 3074, Lismore, New South Wales 2480 Australia

**Keywords:** Employment, Ageing, Workforce, General practice

## Abstract

**Background:**

Work ability (WA) is an indication of how well someone’s health, skills and experience match current job demands. The aim of this study was to ascertain whether the work ability model can provide a useful explanatory framework to understand some elements of sustainable employability (SE) amongst GPs.

**Methods:**

A thematic analysis of 19 in-depth interviews with GPs in the Northern Rivers region of NSW, Australia, was conducted and formed the basis for a qualitative validation of the work ability model.

**Results:**

In order to provide a more comprehensive reflection on the factors and dynamics found to underpin work ability amongst ageing GPs required the creation of specific subcategories within the WA model. Additionally, new themes relevant to general practice also emerged from the data. The analyses revealed a set of important, new factors and relationships that required additions and refinements to the original model, in order to fully explain sustainable employability in this GP sample. These new emerging themes that required model extension were ‘Work-life balance and lifestyle’, ‘Extended social community’ and ‘Impact of gender’.

**Conclusion:**

While the WA model provides a basic explanatory framework for understanding some elements of sustainable employability amongst GPs, a revision of the current model has been proposed to sufficiently describe the factors impinging on sustainable employability in this group. The extended model can potentially be used for addressing workforce planning issues and to assist in programme design to promote sustainable employability amongst GPs and could potentially be translated to other health professional groups.

## Background

Australia has a workforce shortage of general practitioners (GPs), particularly in rural and regional areas [1, 2]. This phenomenon is reflected globally, in countries including England, Canada, USA, India, Israel and South Africa [3–8]. As GPs are responsible for providing primary care services [9], any deficit in their numbers has a significant potential impact on access to basic medical services and follow-up care [10].

In areas of workforce shortages, GPs are required to work longer hours, often on their own or in a small practice, and at the extremes of their scope of practice [11]. One of the most commonly noted issues for rural and regional doctors is the difficulty in accessing locum support that is timely, affordable or of an adequate quality [11]. Additionally, access to continuing professional development (CPD), including procedural upskilling or specialising can be also difficult to obtain in areas outside major towns and cities [11]. This is a barrier to sustained interest and challenge for GPs and can, together with issues of unsustainable work demands, lead to burnout and early retirement [12]. A further issue is the ageing of the existing GP workforce. Approximately 30% of the GP population in NSW is aged over 55 years and approximately 25% Australia-wide [13], with the average age being 53 years [14]. Not only are rural and regional GPs on average older than their urban counterparts, as a population they also retire at a younger age [12].

Increasing workforce supply is inherently a multifactorial challenge, necessarily entailing both increased recruitment and training of new GPs, of which experienced GPs are an integral part, as well as strategies aimed at minimising early retirement from general practice. It is an important aspect to keep experienced GPs in practice in order to continue to provide healthcare to rural communities as well as to facilitate the training of future generations of doctors. GP recruitment, migration and retention in rural and regional areas are important national matters if shortages are to be addressed [15]. This should necessarily involve the identification of appropriate and effective incentives, as well as strategic efforts directed at addressing barriers and facilitators identified through consultation with GPs.

**Fig. 1:**
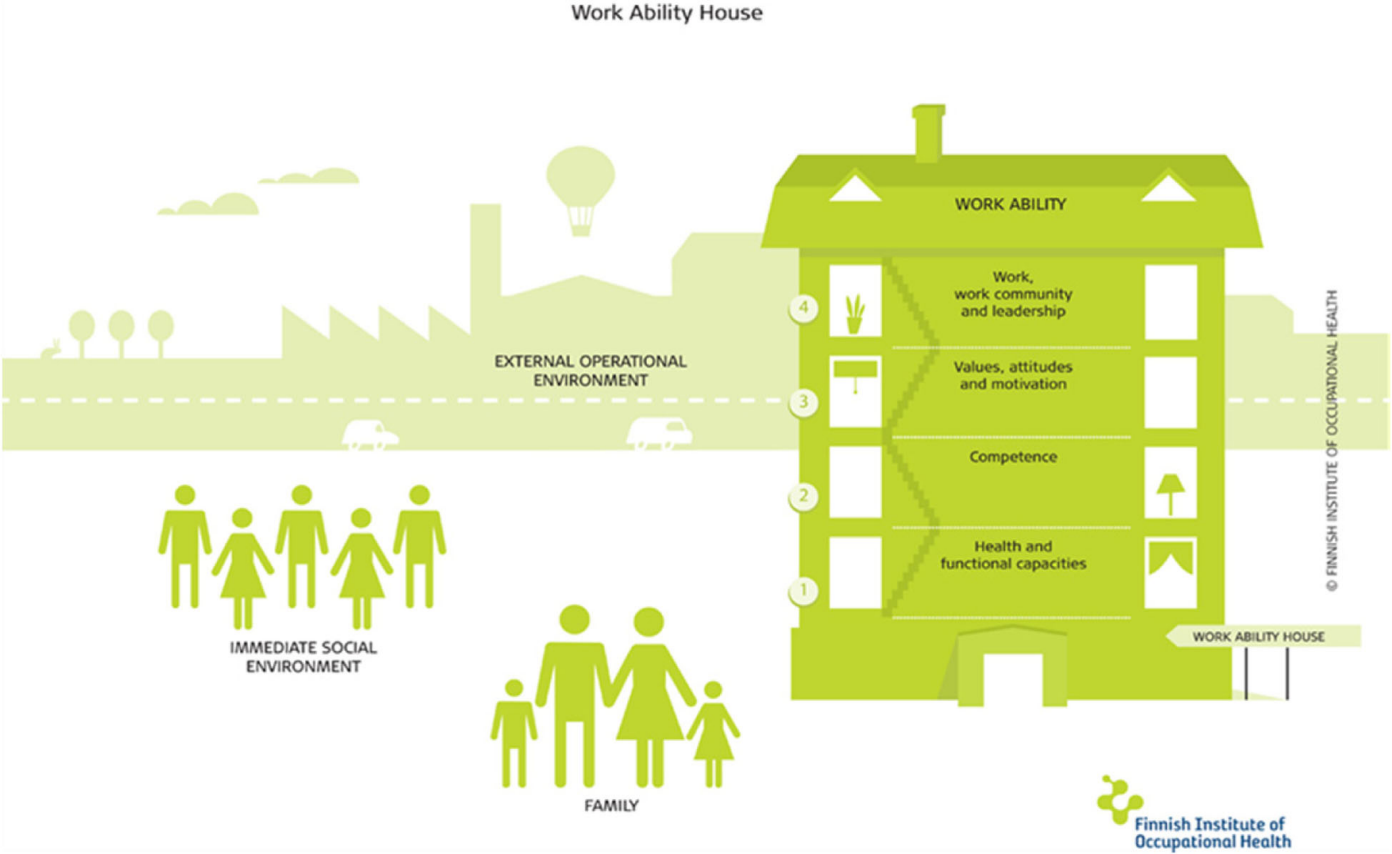
Work ability model (Finnish Institute of Occupational Health, 2014) [17]

The work ability (WA) model has historically been used to explain and explore retirement and long-term employability. The WA model was developed in the 1980s at the Finnish Institute of Occupational Health as an instrument to predict retirement age by analysing the interactions of various factors that affect work ability [16]. The model encompasses the resources of the individual, the external factors related to their work, the environment outside of their work and how these factors relate to an individual’s workability. The model has been visually depicted as a house, with four interconnected floors, and a surrounding environment to illustrate the interactions of all of these elements (Fig. 1).

The work ability concept blends well with the sustainability movement. People, organisations and governments are increasingly aware that employment goes beyond having a job at one point in time, but rather that we need to think about sustainable employment [17]. Van der Klink and colleagues have defined SE as follows: ‘Sustainable employability means that, throughout their working lives, workers can achieve tangible opportunities in the form of a set of capabilities. They also enjoy the necessary conditions that allow them to make a valuable contribution through their work, now and in the future, while safeguarding their health and welfare. This requires, on the one hand, a work context that facilitates this for them and on the other, the attitude and motivation to exploit these opportunities’ [18]. While the capability approach is not synonymous with workability, it is important to note the definition here to place the SE concept into a wider context. While the WA model has historically been used to explain and explore retirement and long-term employability, this may not be the same as sustainable employability. The International Standards Organisation has recently released a guideline on Sustainable employability (SE) for organisations [19]. In this guideline, SE for the individual is defined as ‘the long-term capability to acquire, create and maintain employment, through adaptation to changing employment, economic and personal conditions throughout different life stages’. Workability can be seen as an element of sustainable employability at one point in time and can also be used as a proxy measure to measure sustainable employability [20]. In a recent study amongst 49 content experts, 97% of participants agreed that workability can be used as a proxy measure to measure sustainable employability [20]. Based on the above, we propose the following definition of sustainable employability, in order for it to be more measurable in practice: ‘Sustainable employability refers to a person’s ability to gain or maintain quality work throughout their working lives, while

- having the motivation to conduct quality work
- maintaining good health and wellbeing and
- having the opportunity and the right work context
- co-creating value on personal, organisational and community level
- being able to transfer skills, knowledge and competencies, to another job, company or other future roles’

The WA model does not necessarily have a future component in the model itself but it may lend itself to categorising and exploring factors influencing sustainable employability. Demonstrating this in the case of general practice, it can help us explore how GPs can ‘recycle’ current knowledge, skills and abilities for use in future roles such as teaching or GP advocacy work. It can also help us understand how GPs maintain a work-life balance to sustain a busy demanding career in general practice.

More evidence is needed to demonstrate the effectiveness of SE interventions for ageing workers [21, 22]. The purpose of this study was to ascertain whether the WA model can provide a useful explanatory framework to understand sustainable employability amongst GPs.

## Methods

The current investigation is based on a qualitative analysis of data that was previously collected as part of a larger mixed-method study, described in further detail elsewhere [11]. Participants were recruited via the Northern Rivers General Practice Network (NRGPN), which is a local body representing GPs in the region. The region is a coastal area comprising the far northeast corner of the state. The region depends economically mainly on tourism, with a large number of small towns and comprises localities classified as Small Regional to Medium Large Regional according to the Modified Monash Index [23]. GPs (*N* = 165) received a study package from the NRGPN containing a covering letter from the NRGPN, a participant information sheet, consent form, a reply-paid envelope and an anonymous quantitative survey about early retirement, healthy lifestyle, occupational health and work-related factors [10]. All eligible participants received two reminders 2 and 4 weeks after the initial invitation. GPs who returned a completed consent form for participation in the interview component were contacted to arrange a time and a preferred venue for the interview. Consenting GPs who were unable to participate in a face-to-face interview were interviewed by phone (*n* = 1).

Two interviews were conducted by an occupational health physician, while the remainder of the semi-structured interviews were conducted by Author SP, who is an experienced academic researcher, who was personally known to some of the participants due to her familiarity with the medical professional networks in this geographical area. There were no other people present at the interviews. Interviews lasted approximately 60 min and were mainly undertaken in general practice clinics. A few GPs preferred the interviews to be conducted at a café, their own home, phone or a university location. The semi-structured interview schedule was developed by the authors, and pilot tested with two GPs. Specifically, the questions were asked to explore GPs’ perceptions of the factors which hinder and encourage healthy workforce participation, reasons for choosing to work or not until and beyond traditional retirement age, and to explore current retirement pathways amongst GPs. The interviewer showed and briefly explained the WA model to the participants at the beginning of the interview. Interviews were audiotaped and transcribed verbatim and identifying information was removed.

### Data analysis

NVIVO 10 was utilised to assist with the organisational aspects of the data analysis. A hybrid deductive-inductive thematic analysis approach was used, similar to that described by Fereday and Muir-Cochrane [24]. Initially, two authors independently coded three transcripts and developed a draft coding hierarchy. This was both deductively derived from the WA model [25] as well as inductively generated based on categories arising from in vivo coding. Discrepancies were discussed and the revised coding structure was applied during a first cycle coding of the full data set. This structure was further discussed, refined and expanded by all the authors throughout the coding process, allowing the development of a final coding scheme which was applied the data set during a second cycle coding. A thematic map was developed based on the coded data with the WA model as the organising framework. The alignment with this and the identification of aspects which were not explained within this model were discussed amongst the authors and formed the development of a revised model. Thematic narratives were generated.

## Results

Of the 19 GPs participating, 14 (74%) were male. The average age was 57 years (standard deviation, 12 years). All participants were actively engaged in GP-related employment. Two were in solo practice.

The analyses supported the general applicability of the WA model in understanding SE amongst GPs, in finding that all the elements of the original model were strongly represented in the data. Themes aligned with the model and embodied the fluid and dynamic relationships between the various model components from a general practice perspective. However, in order to provide a more comprehensive reflection on the factors and dynamics found to underpin work ability in this group of GPs, a modification of the WA model was required which entailed the creation of additional subcategories within the model (Appendix). Additionally, new themes relevant to general practice also emerged from the data, which were not reflected in the original model. Hence, the current data set revealed a set of important, new factors and relationships that required additions and refinements to the original model, in order to fully explain sustainable employability in this GP sample. These factors included work-life balance and lifestyle, which both were found to connect to the external and internal environments, the addition of an extended social community to reflect the influence of the wider community within which a GP resides, and the impact of gender.

### Work-life balance and lifestyle

A desire for work-life balance or to pursue a particular lifestyle was a significant influence on where many GPs chose to reside and work. Intrinsically linked to this theme was family. These themes were dynamically interactive and uniquely functioned as a connection between the external environment and the interior of the WA house and all of its floors. Personality and personal characteristics (first floor) determined the GPs desire to pursue a particular lifestyle, and the ability to work in a particular manner drove their education and upskilling (second floor). A balance between lifestyle and work meant improved physical and mental health (first floor) and consequently improved their attitude to work and the perception of intrinsic benefits such as personal fulfilment (third floor). The influence of lifestyle and work-life balance also affected workload (fourth floor):

…we’ve tried to make the job fit the lifestyle rather than the other way ‘round. (GP1)

#### Distance and travelling

GPs identified that pursuing the lifestyle or work-life balance they desired had a significant impact on the time they were required to spend travelling to work, education or social events. GPs described living close to schools for families, near the beach, on a farm, or in a different town to maintain anonymity, which often lead to increased distance and consequent time spent travelling:

When I stopped working at [a clinic], it was because I was spending two hours driving there. (GP13)

As with the theme of work-life balance, this sub-theme interweaved all of the floors of the WA house, as too much travel was found to negatively impact mental and physical health (first floor), and access to education (second floor) in some cases. Prolonged travel times impacted the workplace due to its influence on time management (fourth floor).

### Extended social community

The community in which GPs lived had a direct impact on their work. GPs are recognised members of the community. This was found to have either a positive impact on their work experience, with intrinsic rewards (first floor, third floor):

*…*I contribute to the community here, and that’s where I’m sort of trying to make my mark (GP2)

or a negative impact as community expectations exerted an external pressure to work in a certain fashion (fourth floor):

If you’re in private practice you are basically a slave for your community. (GP14)

Of consideration for many GPs was the lack of anonymity in a small community, the experience of which was mediated by their personality and personal characteristics (first floor):

…I run into patients around and about…that would become a bit of an issue. (GP9)

Finally, the structure of the community itself can influence the type of work a GP is undertaking (fourth floor):

…there’s a lot of people with depression in our community and that’s a major part of their presentation. (GP8)

### Gender

Gender was a complex theme that emerged throughout the interviews. It appeared to have a multilayered impact on the individual, evidenced by gender’s influence on the various floors of the WA model, and also on the GPs’ interaction with their external environment and family. Gender also appeared to exert either an amplification or moderation of the influence of family on the WA model.

Female GPs attracted different presenting problems in their practice with more complex and emotionally loaded problems leading to a higher emotional burden for the GP and longer consultations, which impacted work content (fourth floor) and mental health (first floor):

They might have longer visits. Like [GP name] … she doesn’t see the volume of patients that a few of us see. (GP8)

Some female GPs found that the emotional load from these different presenting problems also necessitated more time for recharge and recuperation, in order to maintain what is perceived as a sufficient level of compassion (first and third floors):

But people tell me as a female GP you get all the tears and smears, and it’s the tears that really drain you at the end of the day. (GP9)

The very specific work content that female GPs often encountered meant varying their education and upskilling (second floor) to match the needs of their patient demographic (fourth floor and external environment). Commitments to family (operational environment) also impacted their access to and time for continued education:

My general practice isn’t really general practice; it’s women’s health, which started when I first came here…I never really ever got to do general practice once I had children because the population came for 1001 Pap smears and other things pertaining to women’s health. (GP13)

In some cases, male GPs avoided a certain kind of work or patient determined entirely by gender:

‘Cause there’s female doctors in the practice, I do less Pap smears… (GP6)

Female GPs also described using different methods to manage their practice (and work within a practice) to their male counterparts, as they defined themselves differently from men. It appeared that women’s identity was still largely tied to their role as a mother and wife:

…but as a woman GP, I was not valued—this is a terrible thing to say—I was not valued in my own practice. And the culture in my own practice, which was an interesting practice, was you’re playing at general practice. You’re a mom; you’re playing at general practice. You’re not a real GP. (GP13)

Hence, it appeared that women’s work ability was more strongly impacted by family than men. Some women felt that they were perceived as less committed than their male counterparts (third floor) due to their apparent prioritising of family commitments and the consequential reduction in (paid) work (fourth floor).

## Discussion

The thematic analysis revealed that much of the data could be broadly categorised according to the WA model, suggesting that the work ability model can be used to explain elements of sustainable employability amongst ageing GPs. Our findings aligned with previous research [26, 27] which identified that the areas of particular relevance to rural and regional GPs were workforce support, rural and regional training opportunities, access to continuing professional development, flexibility in practice ownership, family support, and recognition and remuneration. However, in order to provide a more comprehensive reflection on the factors and dynamics found to underpin work ability in this group of GPs required the creation of specific subcategories within the WA model (Appendix). Quantitative analyses [28, 29] using the work ability index have previously pointed to health and functional capacity (first floor) presenting the highest explanation rate for continued work ability in older workers, followed by work factors (fourth floor). The current thematic analysis supported these findings for GPs.

**Fig. 2:**
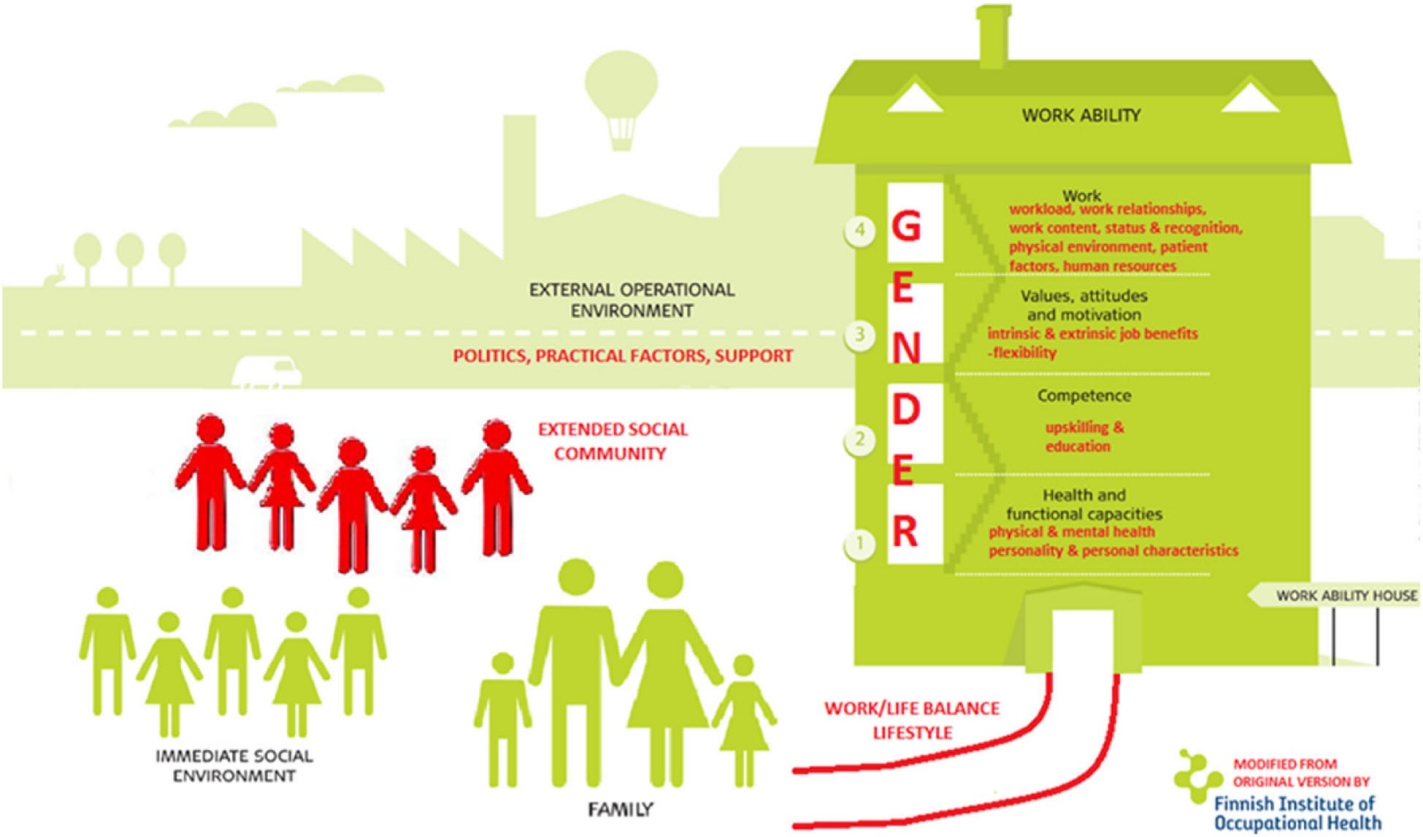
Modified work ability model (modifications marked in red and based on themes identifed in qualitative analyses)

In addition to the specific subcategories, which emerged from the data, entirely new factors were identified: work-life balance and lifestyle, extended social community, and gender. Hence, a modified WA model is proposed to more accurately reflect the components of work ability which forms the basis for sustainable employability in GPs. (Fig. 2).

Work-life balance and lifestyle provided important and previously unidentified connections between the WA House and the external environment. Where and how a GP chose to live impacted the various elements of their work ability and vice versa. This finding supported previous research which has alluded to the importance of work-life balance for GPs [11, 30, 31].

The data also revealed that rural GPs experienced the added dimension of an extended social community not previously included in the WA model. The community in which the GP lived had a direct impact on their work. They felt to be recognised (and ‘public’) members of the community, which for some had a positive effect on their work experience (intrinsic rewards), while others experienced a negative impact with increased work demands and lack of anonymity. Further research would be beneficial to ascertain whether the significance of the extended social community could be extrapolated to other (health) professional groups.

Gender was the third emergent theme that could be added as an element to the original WA model to explain GPs’ sustainable employability. Previous research into the impact of gender on work ability has alluded to women professing greater intolerance to some work challenges earlier than men, especially with regard to physical requirements, but demonstrated greater tolerance for jobs requiring a high cognitive demand compared to men [32]. Women have also been found to be exposed to more unfavourable work conditions than men even when they carry out the same work [32], which corroborates the findings of this study.

Gender impacted every floor of the WA ‘House’ and had a moderating or amplifying effect on the influence of family. Women GPs were predisposed by their roles as mothers and caregivers to be more likely to modify their work hours to accommodate family commitments compared to their male counterparts. These findings suggest there may be merit in developing gender-specific sustainable employability frameworks.

### Strengths and limitations

While care should be taken in generalising the current findings to other GP or professional populations, a major strength of this study was the inclusion of a cross section of GPs within a well-defined geographical area of New South Wales. Additionally, the achievement of data saturation and alignment of the current results with previous research lend support to the validity of the findings. Studies of larger GP populations from a variety of regions/communities would provide further empirical validation of this modified model. This study would also be strengthened by the inclusion of GPs not currently engaged in practice, as this may have provided a more accurate picture of the factors contributing to early exit. This should ideally be a focus of further research.

### Impact of research outcomes

This study has revealed the importance of new factors influencing work ability in GPs. It has provided a more comprehensive model for effectively explaining elements of sustainable employability for GPs in the Northern Rivers region of New South Wales. The model can ideally be utilised as a basis for addressing workforce planning issues and to assist in the design of programmes to promote sustainable employability amongst GPs. For example, the WA model can be used as a conversation tool to raise awareness and teach individuals, such as GPs, which factors relate to their own workability and how these factors may influence their own sustainable employability [33].

## Conclusions

This is the first study that has empirically tested the WA model in a general practice population using qualitative methodologies. In our study, we tested whether the WA model may lend itself to categorising and exploring factors, which influence sustainable employability. The WA model can be utilised to understand elements of sustainable employability amongst GPs. However, work-life balance and lifestyle, extended social community and gender were aspects of work ability pertinent to GPs which did not form part of the original model and required inclusion to more accurately reflect the components contributing to sustainable employability amongst GPs.

## Appendix

Themes identified in the current analysis which align with the original WA model.

### WA model first floor—health and functional capacities

*Physical and mental health*: while many GPs stated that their work was not physically taxing, physical health and age were identified as significant factors in the hours they worked (fourth floor) and their continuing work ability. I really don’t view retirement as being...a finite point for me, in terms of age, because this is not a physical job…my brain still works. (GP2)

The lack of physical demands highlighted the importance of mental health, of the ability to manage stress, and the intellectual and emotional challenges of the work they encountered. This is closely interlinked with personality and personal characteristics, and with values and attitudes to work (third floor). I think the first obvious one would be my health, which at the moment is good and fine, but of course if that changed dramatically I could have to leave at short notice... (GP11)

Working as a GP was recognised as having a direct effect on health, which in turn impacted the ability of the individual to work. GPs identified the positive aspects, like health promotion, which increased their work ability, but also the negative impacts of work-related stress. Alcohol intake is limited because you’re on call, so that’s been good—I’d probably have a few drinks every night otherwise, but I only have a drink a couple of days a week now. (GP1)

Work and health had a fluctuant and reciprocal relationship which uniquely connected the first floor with the fourth floor in the WA model. …I know a couple of people who got ill who rightly or wrongly felt that the stress of their job and not looking after themselves well enough [because of the workload] was a key component of them getting unwell… (GP11)

Age and physical and mental health are connected to upskilling and continuing education (second floor). An older or overly stressed GP was less likely to have the desire or capacity to pursue further education and enhancement of their skills.

*Personality and personal characteristics*: the resilience and personality of a GP directly impacted their work ability. It formed the basis of their desire and ability to work as a GP, and interacted with their mental health (first floor). Personality directed motivating factors and intrinsic benefits (third floor) and affected the workplace (fourth floor), shaping interactions with colleagues and patients and attitude to workload. I think it has a lot to do with personality; I think it’s a question of resilience or the lack thereof; so one has to be resilient. I think that personality-wise, doctors generally tend to be fairly obsessional people; they like to get everything just right, and when things don’t go just right, well they get nervous and anxious, and in severe cases, get depressed. (GP4)

### WA model second floor—competence

*Upskilling and continuing education*: the ability to pursue and practice in areas of special interest was identified as an intrinsic benefit (third floor), and having the opportunity to learn new skills impacts the workplace (fourth floor) where the GP is able to use these skills for continued interest and challenge. A GP’s physical and mental health (first floor) influenced their desire and ability to undertake further education and upskilling. Access to and opportunity for continued education and upskilling is therefore an important factor in sustainable employability. …my other interest is Dermatology… so that I can actually get my skills up and perhaps doing a lot of sun cancer medicine type thing (sic). (GP1)

Accessibility to training and education was found to be lacking in some rural centres: The educational opportunities aren’t always great locally… (GP8)

### WA model third floor—values, attitudes and motivation

*Values and attitudes*: the individual’s attitude toward work and their personal values had a considerable impact on their work ability and linked closely to personality and personal characteristics (first floor). Some older GPs identified a work ethic or attitude that they felt was a product of their era that kept them working. I always intend to work till I die...So I’ll be working forever. (GP1)

This work ethic kept GPs engaged in their careers and accessing education and upskilling (second floor) to maintain their competency and work ability. …people don’t have to work as hard as we did then… (GP15)

The younger generation of GPs represented a shift in thinking toward a more balanced view of work and lifestyle that was reflected in their work habits, with many working part-time (fourth floor) and putting more emphasis on home and family life (external environment) than their older counterparts. This change in attitude also reflected a wider change in societal values indicating the influence of the external environment on this floor of the WA model. …the young GPs coming through have no intention of ever working full-time. I think that’s extremely sensible. (GP7)

*Intrinsic job benefits*: potent motivating factors were the intrinsic rewards that GPs received from working. These included the daily challenge of diagnosing and managing patients (fourth floor) and the opportunity to explore special interests (second floor). I think it [acute hospital work] is a great extension to general practice work. I find that for me you know, it’s challenging…I’m not just looking at my general practice day; I’m also looking at my acute hospital days. So it is something, there is a motivational force in a way. (GP2)

Furthermore, there was the positive reinforcement of helping a person to change their health for the better, as one GP said: …that’s the feedback that you need to keep going; you’ve got to feel good about what you do… (GP4)

This sense of fulfilment and reward aligns with personality and personal characteristics and has a positive connection and impact on mental health (first floor).

*Extrinsic job benefits*: monetary reward and the consequent ability to live a desired lifestyle or satisfy financial requirements was a commonly identified incentive to continue working as a GP. This connects the external environment factors of family, via the need to provide for them financially, and the work-life balance and lifestyle theme which connects the interior to the exterior of the house of the WA model. Monetary reward also tied in closely with recognition and status (fourth floor), and to education and upskilling (second floor) to enable a GP to pursue more lucrative special interests. We all want to be paid better, there’s no doubt that general practice is the most poorly paid medical specialty… (GP7)

One GP spoke in plain terms that financial reward was not just about income for them, highlighting the connection with personality and personal characteristics (first floor): …people need to feel valued…you don’t get paid as much…you know, it ranks less. (GP17)

*Flexibility*: within the theme of extrinsic job benefits is flexibility, both in the workplace and in the work the GP is able to do. …flexibility I think is really important to keep people in the workforce. (GP6)

Being able to modify work hours or areas of interest was identified as an important consideration in continuing to work in a particular practice or community. This overlaps with many other themes of the WA model. The need to be flexible reflects a personal characteristic, and the satisfaction of this need would positively impact mental health, and potentially physical health if flexibility allowed the GP to engage in more active pursuits (first floor). Flexibility allows for the pursuit of further education (second floor). Finally, flexibility has the most influence on workload, work content and even on work relationships as it indicated the ability to compromise (fourth floor). Just by flexibility of the workplace, so that if the only time you can get to the gym is between 11 and 12 o’clock, then as long as you’re doing your hours, go and do that then. (GP11)

### WA model fourth floor—work

*Workload*: The most recurrent and therefore strongest theme identified related to workload. This theme encompassed the hours worked, the additional pressures and stressors of running a practice or the reduction of same through working as a contractor. Excessive workload was frequently identified as a barrier to longevity of working as a GP, while alternatively the ability to be flexible with the hours worked, or to have support in the form of locums and practice nurses, was described as essential to continued working. So I’m working full-time 4 days a week, but also working maybe 2 weekends a month…I also work as a VMO at the local hospital—I see patients every day there. (GP2)

*Work relationships*: many GPs indicated the importance of their interactions with their work colleagues as:

- an incentive to work (third floor);
- a way to alleviate work pressures and increased social connectedness (first floor);
- a way to perform better in the workplace due to added support.
  The other GP that I work with [GP name], we get on really well. We’ve got quite different styles but they work together pretty well. And the rest of the staff, we all get on very well. (GP1)

Work relationships therefore appear to have a direct influence on workload, which in turn, as one GP identified, was an important intrinsic benefit (third floor): I think the number one thing for keeping doctors here is having enough other people to help with the workload. I think that’s the most important thing, it’s more important than money, it’s more important than the actual work environment. (GP1)

*Work content*: having the opportunity to utilise existing skills or to focus on an area of special interest within the scope of their work was an important factor for many GPs in their continuing interest and sense of positive challenge within the workplace. This reflects personality and personal characteristics (first floor) and competency (second floor) as the GP needs to be educated in these skills to be able to practise them. Utilising existing skills or knowledge was also a way for GPs to transition into retirement without having to finish working entirely, for example, as a teacher or mentor for junior doctors and registrars. Finally, there was a sense of personal reward and of recognition of their skills or accumulated knowledge (third floor). …in an ideal world I’d like to be doing public procedural work (GP3)

*Status and recognition*: another aspect of continued work ability was identified as the need for recognition of the importance of the GP role within the healthcare system. This was deemed to be especially desired from work colleagues and the wider community, including governing bodies responsible for legislation; …people would say to me, doctors would say ‘You’re not a real doctor anymore, because you’re not doing emergency, hospital anymore’ so that kind of attitude is not helpful… (GP6)

This theme connects with values and attitudes (third floor) and with personality and mental health (first floor). Recognition and status also reflect the many years of study required to become a competent GP (second floor). …I also think that GPs are under-rewarded or underappreciated from our own medical community. (GP9)

*Physical environment*: the environment in which the GP practised had an impact on the way they worked and even in their continued desire to work. The size of the practice and access to equipment that facilitated their work or areas of interest was described as an important factor of work ability. …Dermatoscope, otoscopes, you know that they are accredited practices with suitable medical equipment…I would struggle to work for a practice that didn’t have that… (GP10)

*Patient factors*: interactions with patients had a significant effect on GPs and their work ability and even on the type of medicine they practised. The additional external environment theme of extended social community connects with patient factors, as particular communities possess a predominance of a certain type of patient (e.g. retirees, farmers, miners) which influences the type of medicine a GP practises. Also patient expectations were identified as a strong influence on how a GP worked, affecting hours worked, on-call availability, and hospital duties. Yes, once you’ve got your patient base—their expectations…an example of that is a really popular local doctor here, and he really wanted to do the part-time thing, but he had thousands of really dependent patients, and you know, and his personal ethic is always to work really hard, and then when he didn’t want to work hard there was this sort of [backlash] (GP2)

Patient factors also overlap with intrinsic benefits (third floor), as one GP said: ...I have good relationships with the patients and I get a lot of enjoyment out of that. (GP3)

Positive patient interactions also impact mental health (first floor). Finally, GPs pursued further education and upskilling to meet the needs of their patient base (second floor).

*Human resources*: closely associated with workload and workplace interactions was the availability of human resources to support GPs in the workplace. This included locum support to enable GPs to take leave or to distribute their workload: We’ve had a lot of registrars come through in the past; none of them really wanted to stay… (GP1)

The inclusion of practice nurses and allied health practitioners helped coordinate care and further share some of the burden of patient management. I’ve worked in just about every capacity I can think of, from solo up, but since this concept of team care, and having practice nurses, and practice managers has started, we’re no longer a harassed individual in a single room, trying to solve the world’s problems on its (sic) own. (GP7)

### WA model—operational environment

*Family*: the GP’s family was consistently identified as one of the most powerful factors in work ability. Family affected almost every level in the WA model house. Family pressures or rewards had a direct impact on stress and mental health, as well as physical health, for example through physical activity with the children (first floor).

Family constraints influenced the ability for a GP to have time and access to further education and training (second floor), or alternatively influenced the type of training as the GP structured their work around their family. Financial security for family was an extrinsic benefit and motivating factor (third floor). Finally, family influenced hours worked and even location of the workplace (fourth floor). ...My youngest is 15 and will leave or finish school in 3 years. And at that stage we plan to leave [inland village], not because of the job but because of those things. (GP1)

*Social community*: encompasses relatives, friends, and acquaintances. GPs identified the overlap between the work they undertook and the relationships they formed within their community. It correlates with intrinsic benefits and motivating factors (third floor) and physical and mental health (first floor). I have a medical role with those, with the <sporting team>, and you know, I’ve got good friends in this area. (GP8)

### WA model—society

***Politics****:* the ‘red-tape’ and paperwork associated with general practice was identified as a significant negative factor in work ability, impacting motivation (third floor), and workload (fourth floor). …I think it’s a pain, and I would think it would put people off being a general practitioner. We’re just like scribes. (GP15) …every time the government gets into, does something, it makes the administrative work more. They say that they cut the red tape down, and they’ve made it longer. (GP5)

Government policies and bureaucracy were often seen as counterproductive and even antagonistic: I think the government…when it suits them they love GPs and when it doesn’t suit them they attack them. (GP11)

There were reciprocal effects on the mental health, and personality and personal characteristics (first floor) played a part in how strongly GPs felt impacted by politics and whether they took a political role for themselves.

*Operational factors*: since the global financial crisis (GFC) some GPs found that the external impact on their finances (third floor) had affected their workload (fourth floor). …I’ve spoken to the GPs who’ve said they need to stay in [the workforce] longer to top up their super... (GP4)

Financial independence (third floor) was identified as a reason a GP might retire early from work: …they retired at 60 because they had that nice, juicy index pension, so they’re comfortably off. (GP12)

GPs also spoke of the lack of flexibility (third floor) in reducing their hours, or changing to a teaching role (fourth floor), as a deterrent to continuing working. ...just your medical indemnity insurance, that if you’re working less hours it needs to step down…They’re [governing bodies] addressing it… (GP4)

*Support*: GPs reported both positive and negative experiences with regard to support from external bodies, for example the Royal Australian College of General Practitioners (RACGP). Support was beneficial in the areas of providing locums, staff, practice infrastructure, and advocating for GPs politically (fourth floor). GPs indicated that support from advocating bodies had one of the lowest levels of influence on their work ability.

## Abbreviations

CPD: Continuing professional development
GP: General practitioner
NRGPN: Northern Rivers General Practice Network
SE: Sustainable employability
USA: United States of America
WA: Work ability
